# A short version of the *Food Cravings Questionnaire—Trait*: the FCQ-T-reduced

**DOI:** 10.3389/fpsyg.2014.00190

**Published:** 2014-03-04

**Authors:** Adrian Meule, Tina Hermann, Andrea Kübler

**Affiliations:** Department of Psychology I, Institute of Psychology, University of WürzburgWürzburg, Germany

**Keywords:** food craving, Food Cravings Questionnaire, psychometric properties, validity, reliability, body mass index, dieting success, food-cues

## Abstract

One of the most often used instruments for the assessment of food cravings is the *Food Cravings Questionnaire* (FCQ), which consists of a trait (FCQ-T; 39 items) and state (FCQ-S; 15 items) version. Scores on the FCQ-T have been found to be positively associated with eating pathology, body mass index (BMI), low dieting success and increases in state food craving during cognitive tasks involving appealing food stimuli. The current studies evaluated reliability and validity of a reduced version of the FCQ-T consisting of 15 items only (FCQ-T-r). Study 1 was a questionnaire study conducted online among students (*N* = 323). In study 2, female students (*N* = 70) performed a working memory task involving food and neutral pictures. Study 1 indicated a one-factorial structure and high internal consistency (α = 0.94) of the FCQ-T-r. Scores of the FCQ-T-r were positively correlated with BMI and negatively correlated with dieting success. In study 2, participants reported higher state food craving after the task compared to before. This increase was positively correlated with the FCQ-T-r. Hours since the last meal positively predicted food craving *before* the task when controlling for FCQ-T-r scores and the interaction of both variables. Contrarily, FCQ-T-r scores positively predicted food craving *after* the task when controlling for food deprivation and the interaction term. Thus, trait food craving was specifically associated with state food craving triggered by palatable food-cues, but not with state food craving related to plain hunger. Results indicate high reliability of the FCQ-T-r. Replicating studies that used the long version, small-to-medium correlations with BMI and dieting success could be found. Finally, scores on the FCQ-T-r predicted cue-elicited food craving, providing further support of its validity. The FCQ-T-r constitutes a succinct, valid and reliable self-report measure to efficiently assess experiences of food craving as a trait.

## Introduction

Craving refers to an intense desire or urge to use a substance and frequent experiences of craving are a core feature of substance use disorders (Tiffany and Wray, 2012). However, the term craving does not only refer to drug-related, but also to other substances like food or non-alcoholic beverages (Hormes and Rozin, 2010). Accordingly, food craving refers to an intense desire or urge to eat specific foods of which chocolate is the most often craved one among other highly palatable foods (Weingarten and Elston, 1990, 1991). Cultural differences have also been noted: for example, a preference for savory over sweet foods in Arabian countries or the presence of rice cravings in Asian countries (Hill, 2007; Komatsu, 2008). It is the intensity and specificity that differentiates food craving from feelings of plain hunger (Hill, 2007). Although food craving and hunger often co-occur, an energy deficit is not a prerequisite for experiencing food craving, that is, it can also occur without being hungry (Pelchat and Schaefer, 2000). Food craving experiences are common and reported by the majority of adults. That is, although more intense and more frequent experiences of food craving are associated with overeating, they do not necessarily reflect abnormal eating behavior and are not synonymous with increased food intake (Hill, 2007).

The sight, smell, or taste of food and food-cues elicit so-called cephalic phase responses, which prepare the organism for food ingestion and are associated with increases in craving for those foods (Nederkoorn et al., 2000). Physiologically, those responses involve increases in salivary secretion, cardiovascular activity (e.g., heart rate and blood pressure), body temperature, electrodermal activity, and respiration (Vögele and Florin, 1997; Nederkoorn et al., 2000; Legenbauer et al., 2004). Yet, attempts to measure craving objectively, for example based on physiological data, have been criticized for being unspecific and subjective self-report seems the only viable assessment modality (Shiffman, 2000). The term craving is somewhat vague and often subjects are asked to indicate on a one-item rating scale how strong they crave or desire a specific object. Therefore, there is a need to assess craving as a multidimensional construct with standardized questionnaires instead of single questions. This is particularly important in non-English speaking countries because there is no simple equivalent expression for craving (Hormes and Rozin, 2010).

Several self-report measures for the assessment of food craving have been developed such as the *Food Cravings Questionnaires* (FCQs; Cepeda-Benito et al., 2000a,b), the *Attitudes to Chocolate Questionnaire* (ACQ; Benton et al., 1998; Müller et al., 2008), the *Orientation toward Chocolate Questionnaire* (OCQ; Cartwright and Stritzke, 2008; Rodgers et al., 2011), the *Food Craving Inventory* (FCI; White et al., 2002; Komatsu, 2008; Jáuregui Lobera et al., 2010; Nicholls and Hulbert-Williams, 2013), and the *Questionnaire on Craving for Sweet or Rich Foods* (QCSRF; Toll et al., 2008). Each of these measures represents a different approach to the craving construct. Both the ACQ and OCQ are designed to measure cravings specifically related to chocolate and emphasize the relationship between craving and feelings of guilt (Benton et al., 1998) or the conflict between approach and avoidance inclinations during the experience of craving (ambivalence model; Cartwright and Stritzke, 2008). The FCI measures cravings related to different classes of food (high fats, sweets, carbohydrate/starches, fast-food fats; White et al., 2002). The QCSRF measures the intensity of craving for sweet or rich foods with a mixture of questions referring to momentary craving, but mainly to craving during the past week (Toll et al., 2008). Therefore, all of these instruments assess habitual cravings related to specific kinds of food and are restricted to certain dimensions of food cravings.

As opposed to these questionnaires, the FCQs were constructed to assess craving for a variety of foods, without confining them to certain categories or specific foods, as for example chocolate. Furthermore, the FCQs cover behavioral, cognitive and physiological aspects of food cravings. Finally, the FCQs combine two versions that measure current and habitual food cravings. Therefore, the FCQs are the only currently available food craving questionnaires that (1) do not refer specifically to chocolate or similar, (2) assess food cravings on a multidimensional level, and (3) measure food cravings as trait and state. Moreover, there is evidence that the FCQs can be used easily as a measure for specific cravings, for example by replacing references to food with references to chocolate (Rodriguez et al., 2007).

The FCQs are arguably the most extensively validated food craving measures and are available in English (Cepeda-Benito et al., 2000b), Spanish (Cepeda-Benito et al., 2000a), Dutch (Franken and Muris, 2005; modified version from Nijs et al., 2007), Korean (modified version from Noh et al., 2008), and German (Meule et al., 2012a). The trait version of the FCQs (FCQ-T) consists of 39 items and items are scored on a 6-point scale ranging from *never* to *always*. Its original form comprises nine subscales measuring food cravings as (1) intentions to consume food, (2) anticipation of positive reinforcement, (3) relief from negative states, (4) lack of control over eating, (5) preoccupation with food, (6) hunger, (7) emotions, (8) cues that trigger cravings, and (9) guilt (Cepeda-Benito et al., 2000a,b). However, the factorial structure could only partially be replicated in subsequent studies in obese individuals, in a study using the chocolate-adapted version, and in a study using the German version (Rodriguez et al., 2007; Vander Wal et al., 2007; Meule et al., 2012a; Crowley et al., in press). Specifically, results yielded fewer factors in those studies, that is, eight, seven, or six subscales (Rodriguez et al., 2007; Vander Wal et al., 2007; Meule et al., 2012a; Crowley et al., in press). Internal consistency of the total scale is very high (α > 0.90) across different versions and samples (Cepeda-Benito et al., 2000a,b, 2003; Nijs et al., 2007; Rodriguez et al., 2007; Vander Wal et al., 2007; Moreno et al., 2008; Meule et al., 2012a).

The state version of the FCQs (FCQ-S) consists of 15 items to be scored on a 5-point scale ranging from *strongly disagree* to *strongly agree*. Its original form comprises five subscales measuring current food craving in relation to (1) an intense desire to eat, (2) anticipation of positive reinforcement, (3) relief from negative states, (4) lack of control over eating, and (5) hunger (Cepeda-Benito et al., 2000a,b). Like for the trait version, subscales could only be partially replicated in a sample of obese individuals and using the German version (Vander Wal et al., 2007; Meule et al., 2012a). Again, internal consistency for the total scale is usually high (α > 0.90) (Cepeda-Benito et al., 2000a,b; Nijs et al., 2007; Vander Wal et al., 2007; Moreno et al., 2008; Meule et al., 2012a).

Scores on the FCQ-T are positively correlated with BMI, scores on the disinhibition subscale of the *Eating Inventory*, eating disorder psychopathology, food addiction symptoms, and low dieting success (Cepeda-Benito et al., 2000a,b, 2003; Franken and Muris, 2005; Meule et al., 2011b, 2012c; Meule and Kübler, 2012). Accordingly, FCQ-T scores are elevated in patients with bulimia nervosa, binge eating disorder, and obesity (Abilés et al., 2010; Van den Eynde et al., 2012). Thus, higher scores on the FCQ-T are associated with higher scores on various self-report measures related to overeating and with higher body mass in both non-clinical and clinical samples.

Scores on the FCQ-S are positively correlated with length of food deprivation, current negative affect, and are sensitive to food intake and food-cue exposure (Cepeda-Benito et al., 2000b, 2003; Vander Wal et al., 2005, 2007; Meule et al., 2012a,d, in revision; Meule and Kübler, in revision). Thus, unlike scores on the FCQ-T, scores on the FCQ-S are indeed affected by momentary physiological and psychological states and environmental circumstances. Further support for a valid differentiation between state and trait food cravings is provided by the FCQs' retest-reliabilities: 3-week retest-reliability for the FCQ-T is high [*r*_(*tt*)_ > 0.80] while it is, expectedly, substantially lower for the FCQ-S [*r*_(*tt*)_ < 0.60] (Cepeda-Benito et al., 2000b; Vander Wal et al., 2007; Meule et al., 2012a). Yet, the FCQ-T is also sensitive to changes in eating behavior: decreased scores on the FCQ-T can be observed after bariatric surgery and behavioral weight-loss treatment in obese individuals (Batra et al., 2013; Rieber et al., 2013; Giel et al., in press).

The FCQ-T and FCQ-S are not independent from one another. For example, scores on the FCQ-T and FCQ-S are weakly positively correlated and, accordingly, scores on FCQ-S are also associated with measures of overeating, but not as consistent as scores on the FCQ-T (Cepeda-Benito et al., 2000b, 2003; Moreno et al., 2008; Meule et al., 2012a; Van den Eynde et al., 2012). One explanation for this could simply be that individuals with more frequent food craving experiences (i.e., “high trait cravers”) have a higher probability to experience craving in general and, thus, it is more likely that they coincidentally experience craving during data collection. Another possibility could be that completing eating-related questionnaires facilitates current food craving, particularly in high trait cravers. Beyond the fact that sometimes correlations between absolute FCQ-T and FCQ-S scores can be observed, it has been found recently that *increases* in FCQ-S scores during cognitive tasks involving pictures of palatable foods are positively correlated with FCQ-T scores (Meule et al., 2012d; Meule and Kübler, in revision). That is, it appears that the FCQ-T represents a valid measure for the assessment of susceptibility for food-cue elicited craving, which, in turn, can be assessed with the FCQ-S. This is further supported by a study by Tiggemann and Kemps (2005) who found that scores on the FCQ-T predicted craving intensity when participants were instructed to imagine their favorite food.

Although in some studies subscales of the FCQ-T have been found to be differentially related to other aspects of eating behavior (Moreno et al., 2008, 2009; Meule and Kübler, 2012; Meule et al., 2012a), it appears that many researchers only use its total score, which is reasonable in light of its instable factor structure and very high internal consistency. Thus, and because the FCQ-T represents rather a long self-report measure, the aim of the present study was to develop and validate a short form of the FCQ-T. For this purpose, we chose 15 items of the FCQ-T with the highest item-total-correlations from the German validation study (Meule et al., 2012a).

Two studies were conducted to examine reliability and validity of this reduced form of the FCQ-T (FCQ-T-r). Factor structure was tested in study 1 which was an online questionnaire-based study. As only items with high item-total-correlations were selected, we expected a one-factorial structure with high internal consistency. A positive correlation was expected with BMI and a negative correlation with self-perceived dieting success as a preliminary indication for validity. In study 2, female participants performed a working memory task involving highly palatable food-cues. As scores on the FCQ-T have been found to be positively correlated with current food-cue elicited craving, we expected that scores on the FCQ-T-r would be positively correlated with increases in state food craving during the task and that scores would predict particularly state food craving after, but not before the task. Again, a positive correlation was expected between FCQ-T-r scores and BMI and a negative correlation between FCQ-T-r scores and self-perceived dieting success. Additionally, small-to-large positive correlations were expected between FCQ-T-r scores with self-reported impulsivity, restrained eating, and eating disorder psychopathology, similar to those found in the German validation study of the long version of the FCQ-T (Meule et al., 2012a).

## Study 1

### Methods

#### Food cravings questionnaire—trait—reduced (FCQ-T-r)

As noted above, 15 items with the highest item-total-correlations were selected from the German FCQ-T (Meule et al., 2012a). Items and their corresponding original item numbers (cf. Cepeda-Benito et al., 2000a,b) are displayed in Table 1. Items included in the FCQ-T-r belonged to the original version's subscales *lack of control over eating* (5 items), *thoughts or preoccupation with food* (5 items), *intentions and plans to consume food* (2 items), *emotions before or during food craving* (2 items), and *cues that may trigger food craving* (1 item). That is, the FCQ-T-r does not include items of the original version's subscales *anticipation of positive reinforcement*, *anticipation of relief*, *hunger*, and *guilt*.

**Table 1:** **Factor loadings and item statistics of the *Food Cravings Questionnaire—Trait—reduced* in study 1**.

**Item**	**Original item no.^a^**	**Factor loading**	***M* (*SD*)**	*****r***_itc_**
1. When I crave something, I know I won't be able to stop eating once I start.	2.	0.76	2.81 (1.19)	0.72
[Wenn ich ein starkes Verlangen nach etwas verspüre, weiß ich, dass ich nicht mehr aufhören kann zu essen, wenn ich erst mal angefangen habe.]				
2. If I eat what I am craving, I often lose control and eat too much.	3.	0.77	2.86 (1.20)	0.73
[Wenn ich das esse, wonach ich ein starkes Verlangen verspüre, verliere ich oft die Kontrolle und esse zu viel.]				
3. Food cravings invariably make me think of ways to get what I want to eat.	5.	0.73	2.52 (1.19)	0.68
[Wenn ich ein starkes Verlangen nach bestimmten Nahrungsmitteln verspüre, denke ich ausnahmslos darüber nach, wie ich das bekomme, was ich essen will.]				
4. I feel like I have food on my mind all the time.	6.	0.74	2.28 (1.15)	0.69
[Ich habe das Gefühl, dass ich die ganze Zeit nur Essen im Kopf habe.]				
5. I find myself preoccupied with food.	8.	0.75	2.30 (1.19)	0.70
[Ich ertappe mich dabei, wie ich mich gedanklich ständig mit Essen beschäftige.]				
6. Whenever I have cravings, I find myself making plans to eat.	18.	0.70	3.08 (1.14)	0.66
[Immer wenn ich ein starkes Verlangen nach bestimmten Nahrungsmitteln verspüre, merke ich, dass ich gleich plane etwas zu essen.]				
7. I crave foods when I feel bored, angry, or sad.	20.	0.68	3.15 (1.34)	0.64
[Ich verspüre ein starkes Verlangen nach bestimmten Nahrungsmitteln, wenn ich mich gelangweilt, wütend oder traurig fühle.]				
8. I have no will power to resist my food crave.	25.	0.68	2.87 (1.21)	0.63
[Ich habe nicht die Willensstärke, um meinen Essensgelüsten widerstehen zu können.]				
9. Once I start eating, I have trouble stopping.	26.	0.81	2.54 (1.19)	0.77
[Wenn ich einmal anfange zu essen, fällt es mir schwer wieder aufzuhören.]				
10. I can't stop thinking about eating no matter how hard I try.	27.	0.81	1.82 (1.04)	0.76
[Ich kann nicht aufhören übers Essen nachzudenken, wie sehr ich mich auch bemühe.]				
11. If I give in to a food craving, all control is lost.	29.	0.84	1.87 (1.12)	0.80
[Wenn ich dem starken Verlangen nach bestimmten Nahrungsmitteln nachgebe, verliere ich jegliche Kontrolle.]				
12. Whenever I have a food craving, I keep on thinking about eating until I actually eat the food.	32.	0.79	2.35 (1.17)	0.75
[Immer wenn ich ein starkes Verlangen nach bestimmten Nahrungsmitteln verspüre, denke ich so lange weiter ans Essen bis ich diese tatsächlich esse.]				
13. If I am craving something, thoughts of eating it consume me.	33.	0.76	1.90 (1.09)	0.71
[Wenn ich ein starkes Verlangen nach bestimmten Nahrungsmitteln verspüre, verzehren mich die Gedanken daran diese zu essen geradezu.]				
14. My emotions often make me want to eat.	34.	0.73	2.61 (1.23)	0.70
[Meine Emotionen bringen mich oft dazu etwas essen zu wollen.]				
15. It is hard for me to resist the temptation to eat appetizing foods that are in my reach.	36.	0.63	3.50 (1.17)	0.58
[Wenn sich appetitliche Nahrungsmittel in meiner Reichweite befinden, fällt es mir schwer der Versuchung zu widerstehen sie zu essen.]				

a Original item numbers refer to the 39-item version as displayed in Cepeda-Benito et al. (2000a,b), Meule et al. (2012a). Note that the German items were used in the current studies.

#### Dieting questions and sociodemographic and anthropometric information

Participants were asked to report their age (years), sex (male/female), height (meters), weight (kg), occupation (student/other), and citizenship (German/other)^1^. Dieting status (yes/no) was assessed with a single question (“Are you currently restricting your food intake to control your weight (e.g., by eating less or avoiding certain foods)?”; cf. Meule et al., 2012b). Self-reported dieting success was measured with the *Perceived Self-Regulatory Success in Dieting Scale* (PSRS) which contains three items that are scored on 7-point scales (Meule et al., 2012c). Internal consistency was acceptable (Cronbach's α = 0.64) in the current study.

#### Procedure and participants

Student councils of several German universities were contacted by e-mail and asked to distribute the study's website URL using their mailing lists. Questionnaire completion took approximately 5–10 min. Every question required a response in order to continue. Study period lasted 2 weeks. The website was visited 403 times and 353 individuals started the study. A total of 324 complete datasets were recorded. Data were filtered with the homepage's (www.soscisurvey.de) data quality check method which is based on the time participants spend completing each page. As a result, data of one participant were excluded and, thus, the final sample size was 323. Most participants were women (*n* = 271, 83.9%), students (*n* = 285, 88.2%), and had German citizenship (*n* = 308, 95.4%). One-hundred and thirty-six participants (42.1%) reported to be current dieters.

#### Data analysis

Firstly, it was tested if data met requirements for exploratory factor analysis using the *Kaiser-Meyer-Olkin Measure of Sampling Adequacy* and *Bartlett's Test of Sphericity*. Although a one-factorial structure was expected, we chose to use exploratory factor analysis to reveal if there was an influence of the items' original subscale. Thus, exploratory factor analysis with principal component analysis was calculated and the number of factors was determined with parallel analysis. Items means, standard deviations, and item-total-correlations were calculated for item analysis. Cronbach's α was calculated to evaluate internal consistency. Relationships between FCQ-T-r scores and sample characteristics and dieting success, respectively, were tested with independent *t*-tests (sex, dieting status) and Pearson correlation coefficients (age, BMI, PSRS). All statistical tests are reported two-tailed. Exact *p*-values are reported in case of significant (*p* < 0.05) and marginally significant (*p* < 0.10) tests, except for *p* < 0.001 or *p* ≥ 0.10 (*ns*).

### Results

**Figure 1:**
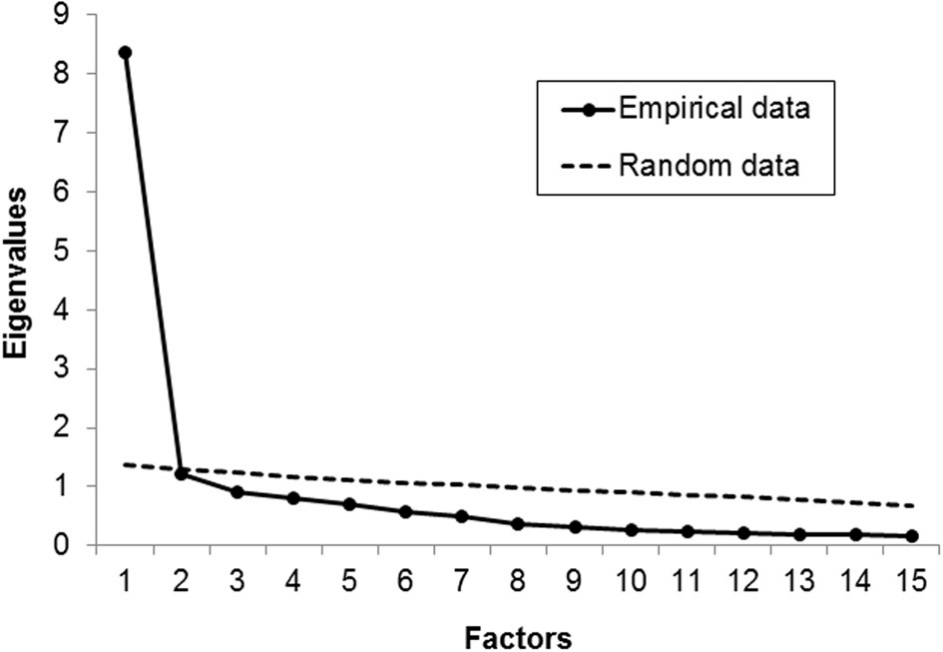
**Scree plot and parallel analysis of eigenvalues in study 1**.

The Kaiser-Meyer-Olkin Measure of Sampling Adequacy (KMO = 0.93) and Bartlett's Test of Sphericity [χ^2^_(105)_ = 3540.35, *p* < 0.001] indicated that the data were adequate for conducting an exploratory factor analysis. Scree plot and parallel analysis clearly indicated a one-factorial structure (Figure 1) which explained 55.85% of variance. Factor loadings and item statistics are presented in Table 1. Internal consistency was high (Cronbach's α = 0.94).

Women had higher FCQ-T-r scores (*M* = 39.48, *SD* = 13.21) than men [*M* = 33.19, *SD* = 11.28, *t*_(321)_ = 3.22, *p* < 0.001] and dieters (*M* = 43.49, *SD* = 13.42) had higher scores than non-dieters [*M* = 34.82, *SD* = 11.62, *t*_(321)_ = 6.21, *p* < 0.001]. Scores on the FCQ-T-r were weakly positively correlated with BMI and moderately negatively with dieting success (Table 2). The correlation between FCQ-T-r scores and BMI was slightly increased when controlling for sex (*r* = 0.18, *p* < 0.001) and was not significant when controlling for dieting status (*r* = 0.08, *ns*). The correlation between FCQ-T-r scores and dieting success was slightly reduced when controlling for sex (*r* = −0.40, *p* < 0.001) or dieting status (*r* = −0.40, *p* < 0.001)^2^.

**Table 2:** **Descriptive statistics of and correlations between variables of study 1**.

***N* = 323**	***M* (*SD*)**	**1**.	**2**.	**3**.	**4**.
1. FCQ-T-r	38.47 (13.11)	–	−0.07	0.15^**^	−0.42^***^
2. Age (years)	24.43 (5.64)		–	0.16^**^	−0.11^*^
3. Body-mass-index (kg/m^2^)	22.00 (3.36)			–	−0.32^***^
4. Perceived Self-Regulatory Success in Dieting	12.14 (3.26)				–

FCQ-T-r, Food Cravings Questionnaire—Trait—reduced.

* *p < 0.05*,

** *p < 0.01*,

*** p < 0.001.

### Conclusion study 1

The FCQ-T-r had a one-factorial structure and high internal consistency. Women and current dieters had higher FCQ-T-r scores, replicating findings from studies using the long version (e.g., Cepeda-Benito et al., 2003; Meule et al., 2012a). Scores were weakly positively correlated with BMI which corresponds to correlations between *r* = 0.10–0.30 in studies with the long version (e.g., Franken and Muris, 2005; Meule et al., 2012a). Moreover, scores were moderately negatively correlated with self-perceived dieting success, which exactly matches the correlation of *r* = −0.42 found in the German validation study of the full version (Meule et al., 2012a).

## Study 2

### Methods

#### Questionnaires

Similar to study 1, participants completed the FCQ-T-r (α = 0.93) and the PSRS (α = 0.63). They also completed the FCQ-S before (α = 0.87) and after the working memory task (α = 0.92). Furthermore, restrained eating was measured with the *Restraint Scale* (RS; α = 0.77) (Herman and Polivy, 1980; Dinkel et al., 2005). Impulsivity was measured with the short form of the *Barratt Impulsiveness Scale* (BIS-15; Spinella, 2007; Meule et al., 2011a) using its total score (α = 0.73) and its subscales representing *non-planning* (α = 0.63), *motor* (α = 0.69), and *attentional impulsivity* (α = 0.64). Eating disorder psychopathology was assessed with the *Eating Disorder Examination—Questionnaire* (EDE-Q; α = 0.95) (Fairburn and Beglin, 1994; Hilbert and Tuschen-Caffier, 2006). Participants also reported the time since their last meal (hours), their age (years), and height and weight were measured for calculation of BMI (kg/m^2^).

#### Stimuli

**Figure 2:**
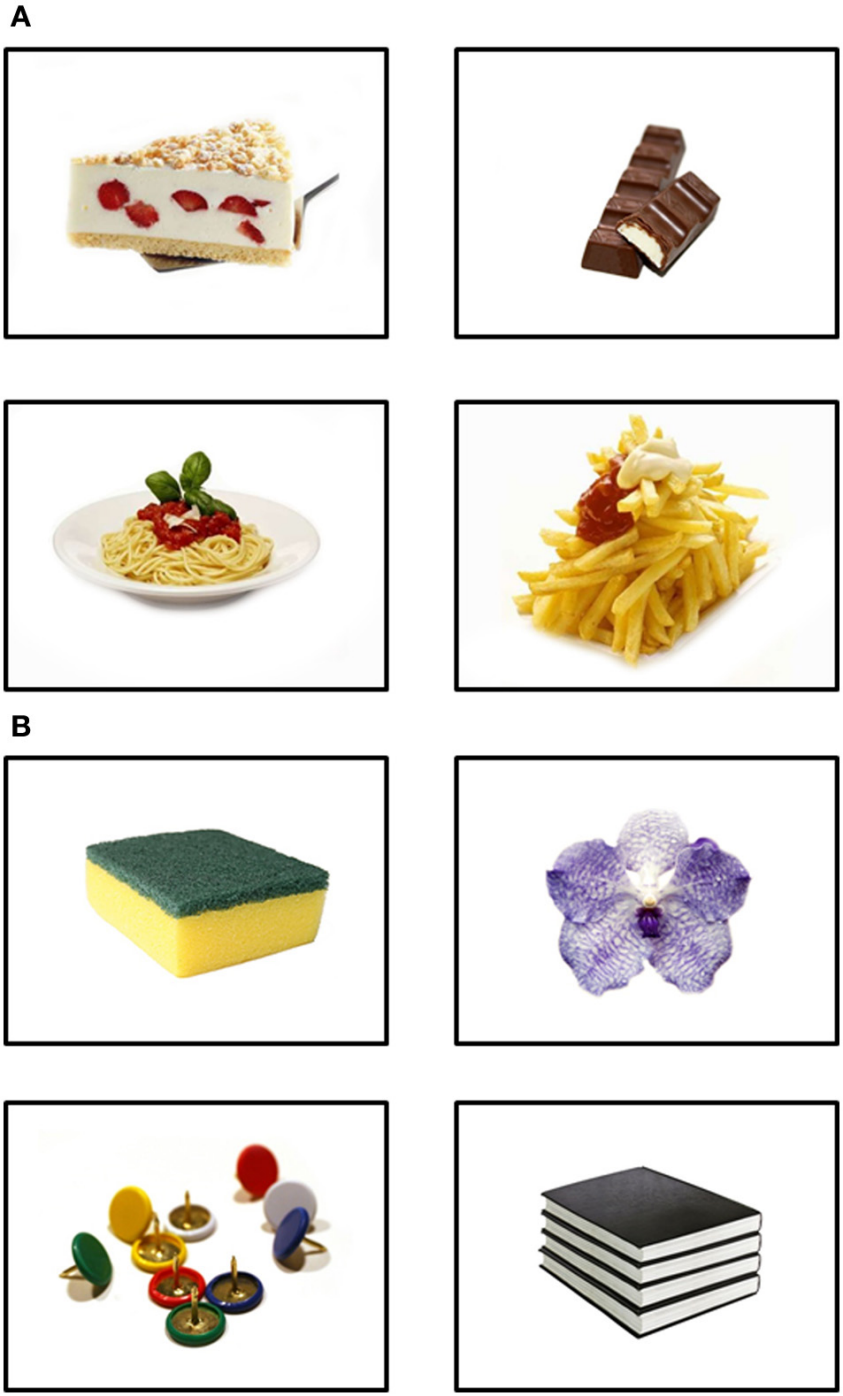
**Exemplary pictures of (A) food and (B) neutral items used in study 2**.

Thirty pictures of high-calorie foods and 30 pictures of neutral objects were selected from the *food.pics* database (cf. www.food-pics.sbg.ac.at; Meule and Blechert, 2012)^3^. Food pictures did not contain meat or fish because vegetarians were not excluded from the study. Exemplary food and neutral pictures are displayed in Figure 2. Picture categories did not differ in visual complexity as based on jpg file size, edge detection, and subjective ratings [all *t*s_(58)_ < 1.48, *ns*]. Food pictures represented food items with a mean calorie content of *M* = 354.77 kcal/100 g (*SD* = 148.01) and mean calories displayed on each image were *M* = 736.95 kcal/image (*SD* = 832.70).

#### n-back task

A working memory task, a version of the *n*-back paradigm, was used in the current study. In this task, stimuli are presented one-by-one and subjects are instructed to press a button whenever a stimulus is presented that is the same as the one presented *n* trials previously (so-called *targets*; in this case, it was a 2-back task). Participants first performed a practice block (14 trials) with numbers and received feedback in case of a false response. The main task consisted of a block with food pictures and a block with neutral pictures (order of blocks was counterbalanced across subjects). Each block contained 120 trials including 30 targets. Each picture was presented four times, but only once as target. Order of trials was pseudo-randomized such that order of target trials was equal in both blocks. Presentation time was 1500 ms or until a response was made. Inter-trial interval (blank screen) was 1000 ms.

#### Procedure and participants

Female psychology students (*N* = 70) were recruited in exchange for course credits. As men less frequently experience food cravings (Cepeda-Benito et al., 2003), they were excluded from the current study to avoid a confounding effect of gender and to ensure comparability to prior studies (cf. Meule and Kübler, in revision). Descriptive statistics of participant characteristics are depicted in Table 3. After signing informed consent, participants completed the FCQ-S and then performed the *n*-back task. After the task, they completed the FCQ-S again, followed by completion of the other questionnaires. Finally, weight and height was measured.

**Table 3:** **Descriptive statistics of trait variables in study 2 and correlations with the *Food Cravings Questionnaire—Trait—reduced* (FCQ-T-r)**.

*****N* = 70****	***M* (*SD*)**	**1**.	**2**.	**3**.	**4**.	**5**.	**6**.	**7**.	**8**.	**9**.	**10**.
1. FCQ-T-r	36. 99 (12.42)	–	−0.16	0.26^*^	−0.35^**^	0.64***	−0.12	0.01	0.40^**^	0.15	0.66***
2. Age (years)	22.00 (3.28)		–	0.07	0.04	−0.07	0.04	−0.04	0.07	0.03	−0.08
3. Body mass index (kg/m^2^)	21.47 (2.82)			–	−0.43***	0.47***	0.06	0.05	0.11	0.11	0.36^**^
4. Perceived Self-Regulatory Success in Dieting	12.36 (3.20)				–	−0.42***	0.03	−0.01	−0.13	−0.06	−0.44***
5. Restraint Scale	12.10 (4.92)					–	−0.14	0.04	0.32^**^	0.11	0.78***
6. BIS-15—non-planning	11.01 (2.29)						–	0.58***	0.05	0.75***	−0.21^#^
7. BIS-15—motor	11.33 (2.39)							–	0.11		
8. BIS-15—attentional	10.41 (2.52)								–	0.57***	0.29^*^
9. BIS-15—total	32.76 (5.06)									–	0.05
10. EDE-Q—total	1.23 (1.02)										–

BIS-15, short form of the Barratt Impulsiveness Scale; EDE-Q, Eating Disorder Examination—Questionnaire.

# *p < 0.10*,

* p < 0.05

** *p < 0.01*,

p < 0.001.

#### Data analysis

As the aim of the present study was to examine the relationships between the FCQ-T-r and state food craving, task performance in the *n*-back task will be reported elsewhere. Relationships between the FCQ-T-r and trait-related variables (age, BMI, PSRS, RS, BIS-15, EDE-Q) were evaluated with Pearson correlation coefficients. Scores on the FCQ-S after the task were compared to scores before the task with a paired-samples *t*-test. Furthermore, a difference score (FCQ-S after the task minus before the task) was calculated for correlational analyses with positive values indicating increases in state food craving during the task. Relationships between the FCQ-T-r and state-dependent variables (hours since the last meal, FCQ-S before the task, FCQ-S after the task, FCQ-S difference score) were evaluated with Pearson correlation coefficients. Finally, linear regression analyses were performed with current food deprivation (i.e., hours since the last meal) and FCQ-T-r scores as predictor variables and FCQ-S scores before the task, after the task, and the difference score as dependent variables. The interaction term of food deprivation × FCQ-T-r scores also was included in all models as relationships between FCQ-T-r scores and state food craving may be moderated by current food deprivation (e.g., maybe higher associations can be found in relatively sated vs. hungry individuals). All statistical tests are reported two-tailed. Exact *p*-values are reported in case of significant (*p* < 0.05) and marginally significant (*p* < 0.10) tests, except when *p* < 0.001 or *p* ≥ 0.10 (*ns*).

### Results

#### Trait variables

Scores on the FCQ-T-r were moderately negatively correlated with self-perceived dieting success. Small-to-large positive correlations were found with BMI, attentional impulsivity, restrained eating, and eating disorder psychopathology. Scores on the FCQ-T-r were not correlated with age, non-planning impulsivity, motor impulsivity, or the BIS-15—total score (Table 3).

#### State variables

**Figure 3:**
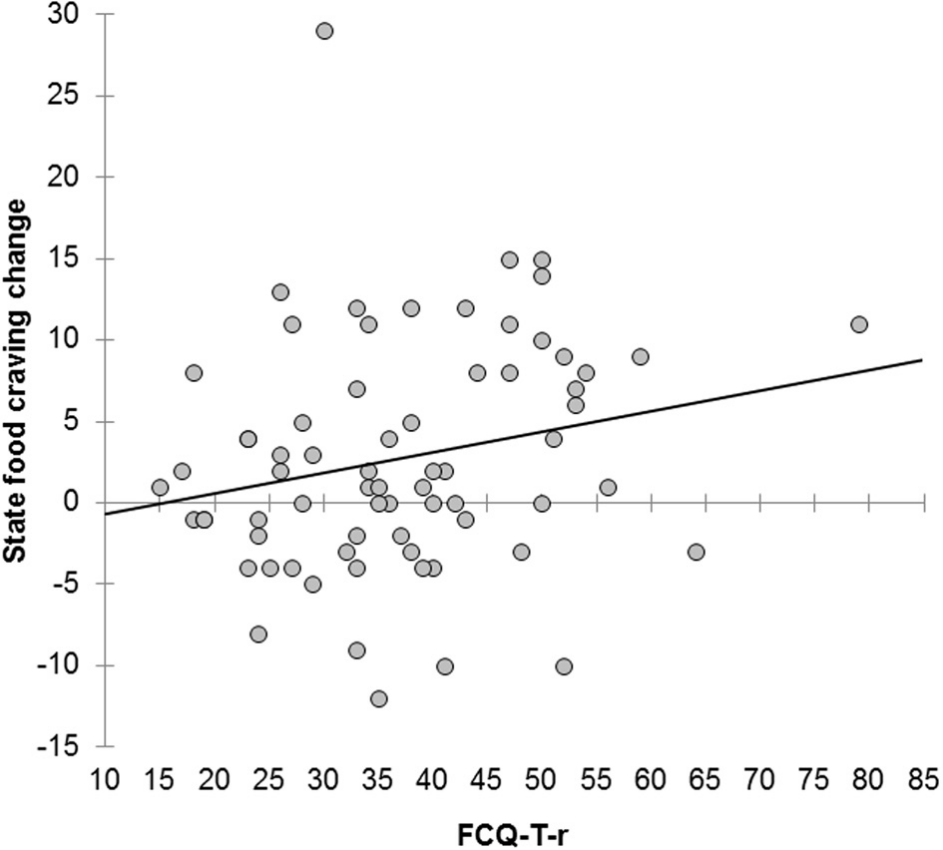
**Scatterplot showing the correlation between scores on the Food Cravings Questionnaire—Trait—reduced (FCQ-T-r) and increases in state food craving in study 2 (*r* = 0.22, *p* = 0.07)**. Exclusion of the two outliers on the top and the right-hand corner did not essentially alter this correlation (*r* = 0.24, *p* = 0.05).

State food craving was increased after the task (*M* = 37.69, *SD* = 11.21) as compared to before [*M* = 34.94, *SD* = 9.35, *t*_(69)_ = 3.21, *p* = 0.002]. Scores on the FCQ-T-r were not correlated with current food deprivation, but were marginally significantly, positively correlated with state food craving before the task and the FCQ-S difference score and significantly correlated with state food craving after the task (Table 4; Figure 3).

**Table 4:** **Descriptive statistics of state variables in study 2 and correlations with the *Food Cravings Questionnaire—Trait—reduced* (FCQ-T-r)**.

*****N* = 70****	***M* (*SD*)**	**1**.	**2**.	**3**.	**4**.	**5**.
1. FCQ-T-r	36. 99 (12.42)	–	−0.02	0.20^#^	0.31^**^	0.22^#^
2. Food deprivation (hours)	4.57 (5.11)		–	0.27^*^	0.21^#^	−0.03
3. FCQ-S—before task	34.94 (9.35)			–	0.77^***^	−0.10
4. FCQ-S—after task	37.69 (11.21)				–	0.56^***^
5. FCQ-S—difference score	2.74 (7.14)					–

FCQ-S, Food Cravings Questionnaire—State.

# *p < 0.10*,

* *p < 0.05*,

** *p < 0.01*,

*** p < 0.001.

#### Regression analyses

Food deprivation, but not FCQ-T-r scores, positively predicted state food craving before the task. Contrarily, FCQ-T-r scores positively predicted state food craving after the task while the influence of food deprivation was only marginally significant. Finally, FCQ-T-r scores, but not food deprivation, marginally significantly, positively predicted increases in state food craving during the task (Table 5).

**Table 5:** **Regression analyses for predicting state food craving by food deprivation and scores on the *Food Cravings Questionnaire—Trait—reduced* (FCQ-T-r)**.

	**FCQ-S before**			**FCQ-S after**			**FCQ-S difference**		
	**β**	***t***	***p***	**β**	***t***	***p***	**β**	***t***	***p***
Food deprivation (hours)	0.25	2.14	0.04	0.20	1.71	0.09	−0.01	−0.12	*ns*
FCQ-T-r	0.18	1.59	*ns*	0.30	2.59	0.01	0.23	1.87	0.07
Food deprivation × FCQ-T-r	−0.20	−1.68	*ns*	−0.13	−1.11	*ns*	0.05	0.43	*ns*

FCQ-S, Food Cravings Questionnaire—State.

#### Conclusion study 2

Again, the FCQ-T-r had high internal consistency and scores were weakly positively correlated with BMI and moderately negatively correlated with self-perceived dieting success. Furthermore, positive correlations were found with attentional impulsivity, restrained eating, and eating disorder psychopathology, which is in line with results of validation studies using the long version (Cepeda-Benito et al., 2000b, 2003; Moreno et al., 2008; Meule et al., 2012a). Overall, state food craving increased during performing the food-related working memory task. Importantly, FCQ-T-r scores specifically were related to increases in state food craving during the task and to increased state food craving after, but not before the task, independent of current food deprivation. Hence, the FCQ-T-r successfully proved to be a measure of the susceptibility to experience food-cue elicited craving, similar to findings of studies employing the long version (Tiggemann and Kemps, 2005; Meule et al., 2012d). It should be noted, however, that the association between FCQ-T-r scores and increases in state food craving is low, which is in line with correlations between *r* = 0.20–0.30 found in prior studies using the full version (Tiggemann and Kemps, 2005; Meule and Kübler, in revision).

## Discussion

The aim of the current study was to develop and validate a short form of the FCQ-T. For this purpose, 15 items with the highest item-total-correlations were selected from the original 39-item version (Cepeda-Benito et al., 2000a,b; Meule et al., 2012a). Expectedly, the FCQ-T-r clearly demonstrated a one-factorial structure in study 1 and had high internal consistency in both studies. The conceptual framework by Cepeda-Benito and colleagues (Cepeda-Benito et al., 2000a,b) acknowledges that food craving represents a multi-faceted construct and, thus, should be measured as such. Specifically, it was identified by the authors that food cravings embrace cognitive, physiological, and behavioral aspects such as (1) concrete intentions and plans to consume food, (2) anticipation of an increase in positive mood or (3) relief from negative states after eating, (4) a loss of control over eating, (5) thoughts or preoccupation with food, (6) hunger, (7) cues that may trigger food cravings, (8) emotions experienced before or during craving, and (9) guilt as a result of (giving into) craving (Cepeda-Benito et al., 2000a,b). Thus, our reduced version of the FCQ-T with only one factor somewhat deviates from the theoretical basis of the original version. It appears that the FCQ-T-r includes essential cognitive and behavioral aspects of food craving experiences such as thinking about food, intending to eat food and losing control over food intake. As a result of their low item-total-correlations, the FCQ-T-r does not include items related to the expected effects of eating such as anticipation of mood enhancing effects after eating and feelings of guilt after giving into cravings. Some researchers have included feelings and cognitions after eating (such as guilt) as an integral component of food craving (Benton et al., 1998; Cartwright and Stritzke, 2008). However, in line with other researchers (May et al., 2012), we would argue that the core components of food craving are its emotional and environmental triggers, mental imagery and cognitive elaboration, and behavioral consequences, that is, searching for and consuming food, which are aspects that are covered by the FCQ-T-r. Furthermore, the FCQ-T-r does not include items for the assessment of habitual hunger. In the FCQ-T, the hunger subscale had the lowest internal consistency (Meule et al., 2012a) and this is also the case in related measures of eating behavior (e.g., Renner et al., 2012). Thus, it may be that it is difficult for individuals to report about their habitual feelings of hunger, which leads to low reliability of such questions. To summarize, although the FCQ-T-r may have the limitation that only a subset of facets associated with food craving is covered, it may at the same time have the advantage that (1) aspects which are difficult to report such as habitual experiences of hunger or (2) aspects that may only be epiphenomenal such as expectations about thoughts and feelings after eating are excluded.

Moreover, although the FCQ-T-r covers fewer facets of food craving, relationships with validity indices were similar to studies that used the full version. That is, FCQ-T-r scores were elevated among women and current dieters, and a small positive relationship was found with BMI and a moderate negative relationship with self-perceived dieting success, replicating prior studies (e.g., Cepeda-Benito et al., 2003; Franken and Muris, 2005; Meule et al., 2012a). In addition, large positive correlations were observed with restrained eating and eating disorder psychopathology which, again, replicates previous studies using the full version (e.g., Cepeda-Benito et al., 2003; Moreno et al., 2008; Meule et al., 2012a; Van den Eynde et al., 2012). With regard to trait impulsivity, correlations were found with the attentional impulsivity subscale only, which is in line with recent findings showing that measures associated with overeating (e.g., the FCQ-T) are particularly related to attentional impulsivity, but only weakly and inconsistently with non-planning or motor impulsivity (Meule, 2013).

Validity was further supported by associations between the FCQ-T-r and self-reported current food craving before and after a food-related working memory task. It has been shown recently that participants reported elevated scores on the FCQ-S after they had performed food-related tasks assessing executive functions such as working memory (Meule et al., 2012d; Meule and Kübler, in revision). In those studies, scores on the FCQ-T were positively correlated with increases of FCQ-S scores during the task. Likewise, in the current study, FCQ-T-r scores were weakly positively correlated with increases in state food craving during a working memory task involving palatable food-cues. We could further demonstrate that FCQ-T-r scores were specifically related to food-cue elicited craving, but not to baseline craving levels. That is, state food craving before the task was predicted by current food deprivation, but not by trait food craving. Contrarily, trait food craving, but not food deprivation, predicted increases in state food craving as well as state food craving after the task.

Shortcomings of the current studies were, firstly, that the majority of participants in study 1, and all participants in study 2, were female students and most of them had normal weight. Thus, interpretation of the current results is limited to this population and results need to be replicated in other samples, particularly men and individuals with obesity or eating disorders. Secondly, as the FCQ-T-r only captures a subset of aspects related to food craving that are included in the full version, additional studies are necessary which directly compare the FCQ-T-r with the FCQ-T to evaluate if the reduced version can be used as a par for par substitute of the full version or if its use leads to different results. However, we would argue that associations with validity measures highly correspond to those found in validation studies of the original version, which does support its use as an alternative measure of trait food craving. This is also supported by a recent study using the Spanish FCQ-T, in which the FCQ-T-r was highly correlated with the full version and the remaining 24 items. Furthermore, correlations with validity measures were similar for the FCQ-T and FCQ-T-r (Rodríguez-Martín and Molerio-Pérez, in revision; see this Research Topic). Thirdly, future studies in which other methods for the assessment of state food craving are used would be valuable in further validating the FCQ-T-r. For example, such studies could include physiological recordings, such as heart rate, during food-cue exposure or ecological momentary assessment. Based on the current findings, scores on the FCQ-T-r should also predict heightened physiological reactivity in response to food-cues or more frequent food craving experiences in daily life, particularly in tempting situations.

To conclude, the current studies showed that the FCQ-T-r has high internal consistency. Relationships with validity measures were comparable to those in studies using the original version. Scores on the FCQ-T-r specifically predicted cue-elicited food craving, providing further support of its validity. Thus, the FCQ-T-r constitutes a succinct, valid and reliable self-report measure to efficiently assess experiences of food craving as a trait.

### Conflict of interest statement

The authors declare that the research was conducted in the absence of any commercial or financial relationships that could be construed as a potential conflict of interest.

## Footnotes

^1^Note that self-reports of some of these variables are potentially biased. For example, height often is overestimated and weight is underestimated (Gorber et al., 2007). However, it has also been found that although such discrepancies exist, reports are usually sufficiently accurate (Bowman and Delucia, 1992). Moreover, anonymous, web-based studies have been found to be advantageous for the assessment of sensitive questions such as body weight (Kroh, 2005; Kays et al., 2012). Thus, we argue that self-reported height and weight probably did not—or only minimally—adversely affect results.

^2^We also performed analyses separately for men and women. Results were similar with regard to factor structure (men: first two empirical eigenvalues 6.90 and 1.58, first two simulated eigenvalues 2.05 and 1.78; women: 8.56 and 1.18, 1.42, and 1.33), explained variance (men: 46.01%, women: 57.07%), internal consistency (men: α = 0.91, women: α = 0.95), and correlations with dieting success (men: *r* = −0.34, *p* = 0.01; women: *r* = −0.41, *p* < 0.001). However, FCQ-T-r scores were unrelated to BMI in men (*r* = −0.00, *ns*), but correlated with BMI in women (*r* = 0.21, *p* = 0.001).

^3^Picture numbers in the food.pics database: 6, 9, 10, 22, 27, 38, 41, 42, 56, 71, 82, 83, 89, 106, 111, 113, 115, 116, 120, 133, 137, 142, 143, 145, 159, 165, 166, 167, 172, 173, 1000, 1001, 1002, 1003, 1004, 1005, 1007, 1009, 1011, 1012, 1013, 1014, 1015, 1016, 1019, 1020, 1021, 1023, 1024, 1025, 1026, 1028, 1029, 1030, 1031, 1032, 1033, 1034, 1035, 1036.
